# An Unjustified Prognosis of the Number of Asbestos-Related Lung Cancer Cases Caused by an Increase in Airborne Asbestos Concentrations as a Result of Removing of Asbestos-Cement Products

**DOI:** 10.1155/2015/264568

**Published:** 2015-03-22

**Authors:** Neonila Szeszenia-Dąbrowska, Beata Świątkowska

**Affiliations:** Nofer Institute of Occupational Medicine, Department of Environmental Epidemiology, The Reference Center for Asbestos Exposure & Health Risk Assessment, Teresy 8, 91-348 Łódź, Poland

We have read the recently published article under an interesting title “Environmentally Related Diseases and the Possibility of Valuation of Their Social Costs” by I. Hajok et al. [1], the main objective of which was “to estimate the risks of the morbidity of the asbestos-related lung cancer in the general population of Poles as the result of increased exposure to asbestos fibers which occurs during the removal and disposal of asbestos-cement products in Poland.”

Contrary to mesothelioma, considered to be a neoplasm specific to environmental exposure to asbestos [2–6], the risk of lung cancer is rarely a subject of analysis in the context of environmental exposure of residents. It is, among others, due to having no features of neoplasm that enable the indication of asbestos as a causative factor, as well as numerous competitive factors of the incidence of this neoplasm. According to the recommendations of the experts of WHO, in the case of this kind of neoplasm, the only practical approach is to use the population attributable fraction (PAF) [7, 8]. Therefore, the published article is even more noteworthy; its contents raise a number of questions and doubts concerning both substantive bases and methodological approach to the analysis.

*Firstly,* the statement* “*increased exposure to asbestos fibers which occurs during the removal and disposal of asbestos-cement products in Poland*”* does not find its confirmation in the published papers. The size of asbestos fibers concentrations during removal of asbestos-cement products was in Poland a subject of numerous measurements taken on various work posts as well as in the atmospheric air. The published results do not confirm the thesis concerning both high concentrations of asbestos fibers during products dismantling as well as, related to these works, a considerable increase in the concentration of asbestos fibers in the atmospheric air, provided safe and legally regulated methods of work are applied [9–13].

*Secondly,* description of the method is limited to a very vague statement: “Taking into consideration the concentration of asbestos fibers in the air of particular administrative area and the number and type of former plants producing asbestos-cement products, as well as standardized incidence rates (SIR) of asbestos-dependent diseases (…) three zones of the country with varying degrees of risk of asbestos-related disease were isolated.” In order to make it possible for the readers to get familiar with the method applied, a reference number [14] was indicated. However, in that paper we do not find answers to any of the elementary questions, inter alia, what does “type of former plants producing asbestos-cement products” mean and how was it determined; there is also no description of the method for calculating standardized incidence rates (SIR) of* asbestos-related diseases*, which turned out to be cases of occupational diseases diagnosed in Poland. Occupational asbestos-related diseases reported in Poland over the years 2001–2009, which the authors used to determine the 3 zones of environmental exposure to asbestos, are a result of exposure in the 70s and 80s of the previous century. During that period concentrations on the work posts ranged from 2 to 8 f/cm^3^, that is, from 2.000.000 to 8.000.000 f/m^3^ of air [15], whereas mean concentrations in the atmospheric air estimated in all provinces of Poland based on the total of 5962 samples for 1634 sampling sites amounted to about 500 f/m^3^ of air, exceeding 1000 f/m^3^ [13] only in few points.

*Thirdly,* based on the description of the method one should presume that in the extrapolation of the incidence of occupational asbestos-related lung cancer (from the area of high doses) in the case of the risk of incidence of lung cancer caused by environmental exposure in the general population (to the area of low doses) occurrence of the same risk was assumed and no coefficient reflecting the size of exposure was applied.

It seems reasonable then to ask what are the substantive grounds for using “incidence ratio” of occupational asbestos-related diseases for the purpose of forecasting the number of lung cancer cases caused by environmental exposure related to dismantling of asbestos-cement products? It should be emphasized that, among individuals who are exposed paraoccupationally and environmentally, the literature has not reported any cases of asbestosis, which constitutes about 60–70% of the total number of cases of occupational diseases induced by asbestos [15, 16].

*In conclusion,* if presented in the article estimation of the risk of incidence of lung cancer associated with exposure to asbestos dust during removal, securing, and dismantling of products, mainly asbestos-cement ones, in the general population of Poland was reliable, the works would contribute to the incidence of 14 thousand new cases of lung cancer. In such a situation the only conclusion is to call for the immediate cessation of the* “Programme for Asbestos Abatement in Poland 2009–2032.”*
